# Hyperthermia-Triggered Doxorubicin Release from Polymer-Coated Magnetic Nanorods

**DOI:** 10.3390/pharmaceutics11100517

**Published:** 2019-10-08

**Authors:** Felisa Reyes-Ortega, Blanca Luna Checa Fernández, Angel V. Delgado, Guillermo R. Iglesias

**Affiliations:** Department of Applied Physics, School of Sciences, University of Granada, 18071 Granada, Spain; felisareyes@ugr.es (F.R.-O.); lunachecaf@ugr.es (B.L.C.F.); adelgado@ugr.es (A.V.D.)

**Keywords:** magnetic nanorod, biocompatible polymer, drug delivery system, doxorubicin, magnetic hyperthermia

## Abstract

In this paper, it is proposed that polymer-coated magnetic nanorods (MNRs) can be used with the advantage of a double objective: first, to serve as magnetic hyperthermia agents, and second, to be used as magnetic vehicles for the antitumor drug doxorubicin (DOX). Two different synthetic methodologies (hydrothermal and co-precipitation) were used to obtain MNRs of maghemite and magnetite. They were coated with poly(ethyleneimine) and poly(sodium 4-styrenesulfonate), and loaded with DOX, using the Layer-by-Layer technique. Evidence of the polymer coating and the drug loading was justified by ATR-FTIR and electrophoretic mobility measurements, and the composition of the coated nanorods was obtained by a thermogravimetric analysis. The nanorods were tested as magnetic hyperthermia agents, and it was found that they provided sufficiently large heating rates to be used as adjuvant therapy against solid tumors. DOX loading and release were determined by UV-visible spectroscopy, and it was found that up to 50% of the loaded drug was released in about 5 h, although the rate of release could be regulated by simultaneous application of hyperthermia, which acts as a sort of external release-trigger. Shape control offers another physical property of the particles as candidates to interact with tumor cells, and particles that are not too elongated can easily find their way through the cell membrane.

## 1. Background

Magnetite (Fe_3_O_4_) and maghemite (γ-Fe_2_O_3_) nanoparticles have found pathways towards application in the biomedical field, in both diagnosis and treatment of different diseases. In addition to the fundamental aspects related to their magnetic properties, the ultimate reason for their use is that they are highly biocompatible and barely or non toxic to humans. Furthermore, they are suited to be functionalized with different compounds according to their sought application [1,2,3,4]. Particularly in the biomedical field, when designed to be injected intravenously, the particles will face the recognition and capture by the macrophages of the immune system [5,6], hence the need to make them biomimetic by coating with, for instance, poly(ethylene glycol) (PEG) [7], dextran [8] or chitosan [9]. Of comparable importance is their transformation into specific-targeting vehicles designed to approach diseased cells and not healthy ones [10]; as examples, we can cite the conjugation with CD44 monoclonal antibodies for specific binding to 4T1 breast cancer cells [11], and the overexpression of folate or biotin receptors in some tumor cells, suggesting the use of folic acid or biotin on the particles for preferential (rarely, exclusive) linkage to cancer cells. The number of possible ligands is certainly large, as has been summarized by Loomis et al. [12].

Many of these applications have been made possible by the variety of methods available for the synthesis of both Fe_3_O_4_ and γ-Fe_2_O_3_ nanoparticles, ranging from “dry” methods as arc-discharge, mechanical grinding, or laser ablation, to “wet” ones based on microemulsions [13], co-precipitation [14], sol-gel [15] and high temperature decomposition of organic precursors [16]. Most investigations related to the use of MNPs in the biomedical field are based on spherical (sometimes rounded, or quasi-spherical) geometries. Non-spherical particles have been less investigated with biomedical applications in focus, although shape effects have been pointed out in the different steps of the drug delivery process, namely, transport in the blood vessels, particle uptake by cells, and particle toxicity [5]. The second one is perhaps the most significant for the purposes of the present work. Nanosized drug carriers have several intrinsic advantages over conventional drug delivery systems (DDS), such as a great loading capacity, protection from degradation, multifunctional moieties, and controlled or sustained release, which reduce adverse effects while enhancing the safety margin of the antitumor agents [17]. Among the nanosized carriers, advantages have been demonstrated in the literature regarding the use of nanorods versus spheres for drug transport and delivery applications. For example, Zhao et al. [18] have shown that rod nanoparticles have a longer residence time in the gastrointestinal tract and a greater capacity to overcoming rapid clearance by the immune system. As a result, this geometry appeared to favor increased bioavailability as compared to spherical nanoparticles. In the review reported by Truong et al. [19], studies are described which demonstrate that non-spherical shaped nanoparticles are the next-generation drug carriers, due to their facile and versatile synthesis methodologies and their flexibility in creating tunable sizes and adaptable surface chemistries. Other authors found that long, elongated particles were captured by tumor cells more efficiently than short rod-like, or spherical ones, but had a similar cytotoxicity [20,21]. Some disadvantages have also been mentioned. Thus, the rigidity of the cell membrane might hinder its ability to engulf particles with a low curvature radius, and hence, endocytosis of elongated particles, leaving mechanical penetration of the membrane as the main mechanism for needle-like particles [22]. This seems to be an open problem, still claiming for further contributions.

The systems proposed here as DDS will have an additional feature on top of their non-spherical geometry, namely, their magnetic (superparamagnetic, in fact) nature. Smart nanocarriers, sensitive to exogenous or endogenous stimuli, such as magnetic fields in the present case, represent an alternative targeted drug delivery method [23]. Non-sphericity brings about shape anisotropy in addition to magnetic (crystalline) anisotropy. The accumulation of the effects of the two sources of anisotropy should provide the nanoparticles with larger coercivity [24], something useful for magnetic hyperthermia, as we describe below.

The magnetic nanorods (MNRs) synthesized in this paper act as smart nanocarriers, being sensitive to both constant or direct current (dc) and alternating (ac) magnetic fields. In the latter case, they can be applied as magnetic hyperthermia (MH) agents. Recall that in MH, the target is to achieve, non-invasively, a localized heating of the volume (for instance, the tumor in which the particles have been previously injected) where the NPs are placed [25,26,27]. This technique has become very popular in recent years as a promising therapeutic modality suitable to be applied in the clinic [28]. Alternating magnetic fields allow a deeper penetration in the body and a less harmful ionizing effect than conventional radio or chemotherapy. Moreover, MH induces tumor cell death through local temperature increase so that thermal effects can also be used as a relevant external stimulus to trigger a drug release from drug-loaded magnetic nanocarriers [29].

In the present study, two different methodologies, namely, hydrothermal and co-precipitation, were used to synthesize Fe_3_O_4_ and γ-Fe_2_O_3_ MNRs, both dispersible in aqueous solution. The hydrothermal method was carried out directly in aqueous media and it had the advantage of producing a homogeneous and well-dispersed suspension of MNRs in water using a low sintering temperature and possibly leading to a pure crystalline phase, depending of the reaction time. Using the co-precipitation approach for producing the maghemite nanorods, it is expected to give homogeneous and phase-pure nanorods in a short reaction time with a narrow size distribution of particles. The magnetic nanorods (MNRs) prepared by both methods are coated with two ionic polymers, namely, poly(ethylene imine) (PEI), and poly(styrene sulfonate) (PSS), using the layer-by-layer (LbL) technique [30].

The MNRs were applied to hyperthermia, but also are envisaged as thermo-responsive carriers, with the aim of producing thermally induced drug release as a result of the alternating magnetic stimulus. The anticancer drug used to be loaded on these MNRs was doxorubicin (DOX), whose efficacy for different kinds of tumors has been extensively demonstrated in the literature [31,32,33], and therefore, it was selected to create a potent thermo-induced triggering DDS. The procedure of combining local heating and optimum release conditions to obtain a higher DOX release lays on the local acidity of the tumor microenvironment [34]. Such tumor acidity offers an advantage for targeted therapy, since it is possible to create a DDS that releases a higher amount of drug in acidic media [35,36], mostly under the action of hyperthermia.

## 2. Methods

### 2.1. Materials

Water (Milli-Q Academic, Millipore, France) solutions of poly(sodium 4-styrenesulfonate) (PSS, *M_w_* = 200,000 g/mol, Sigma Aldrich, (Saint Louis, MO, USA) and poly(ethylene imine) (PEI, *M_w_* = 2000 g/mol, Sigma Aldrich) were prepared with respective concentrations 30 *w*/*v* % and 50 *w*/*v* % on a monomer basis. Ethanol absolute for analysis (Ph Eur grade), sodium acetate anhydrous, and FeCl_3_ 6H_2_O were from Sigma-Aldrich, and used as received. NaH_2_PO_4_ H_2_O was purchased from Scharlau, (Sentmenat, Barcelona, Spain) Phosphate buffer saline tablets manufactured by Sigma-Aldrich were reconstituted by dissolving one tablet in 200 mL water, yielding 0.01 M phosphate buffer (PBS) of pH 7.4, at 25 °C. Glacial acetic acid was from Spectrum (Gardena, CA, USA) and used as received.

### 2.2. Methods

#### 2.2.1. Synthesis of Magnetic Nanorods 

Hydrothermal magnetic nanorods (MNRs) were prepared, starting from a mixture of 0.02 M FeCl_3_·6H_2_O (75 mL) and 0.45 mM NaH_2_PO_4_ (25 mL) solutions. The mixture was transferred to a 100 mL autoclave placed in an oven and heated for 10 days at 100 °C. When the reaction time was finished, the autoclave was cooled down to room temperature and the resulting Fe_2_O_3_ NRs were collected. In order to eliminate excess reactants, the suspension was centrifuged at 12,000 rpm and the solids were dispersed in water. The process of cleaning was repeated three times, and the particles were finally dried at 80 °C overnight. The resulting NPs (denominated H2) were found to be hematite [37], and in order to reduce them and obtain Fe_3_O_4_ MNRs, they were placed in a tube furnace and heated at 300 °C during 3 h with a 30 L min^−1^ N_2_ stream previously bubbled in ethanol. This creates a reducing hydrogen atmosphere (3Fe_2_O_3_ + H_2_ → 2Fe_3_O_4_ + H_2_O). The sample, H2M hereafter, was then cooled down to ambient temperature.

Nanorods prepared by co-precipitation [38] were obtained by adding 20 mmol of FeCl_3_·6H_2_O into 100 mL of deionized water containing 0.5 mL poly(ethylene imine) (PEI). The mixture was heated at 80 °C under magnetic stirring for 2 h, obtaining a precipitate which was then separated by centrifugation and washed several times with a deionized water/ethanol (50/50 *v*/*v*) mixture. The resulting Fe_2_O_3_ NR precursors (J3) obtained was reduced to MNRs (J3M) under the hydrogen atmosphere, as above described. 

#### 2.2.2. Surface Functionalization of MNRs

The functionalization of the MNRs was carried out using the Layer-by-Layer (LbL) technique, or alternating adsorption of polycations and polyanions [30] on the charged surface of the nanorods. The initial layer was prepared as follows: 2.5 mL of an MNRs aqueous suspension (6.5 mg/mL) was adjusted to pH = 8 with 0.01 M KOH aqueous solution. 2.5 mL of PEI (dissolved at a concentration of 80 mg/mL) was then added to this suspension, and the mixture was sonicated with an ultrasound probe for 15 min. The PEI-coated nanorods were then washed with distilled water three times and pellets were collected by centrifugation at 20,000 rpm for 15 min at 25 °C. In order to deposit the second layer, the washed pellet was immersed in 5 mL of a PSS aqueous solution (80 mg/mL), with 15 min ultrasound probe stirring. The PEI-PSS coated nanorods were then washed with water several times, centrifuged at 20,000 rpm for 15 min and dried in an oven at 70 °C for 24 h. Evidence of the presence of both deposited layers was confirmed by electrophoretic mobility measurements and FTIR spectroscopy (Jasco, Tokio, Japan).

#### 2.2.3. Morphology and Size Distribution

The morphology of the MNRs was observed by transmission electron microscopy (TEM) using a LIBRA 120 Plus Carl Zeiss microscope (Zeiss, Oberkochen, Germany) at an accelerating voltage of 60 kV. The samples were prepared by deposition of drops of the corresponding nanoparticle suspension (0.01 mg/mL) over small Cu grids (3 mm diameter), and the solvent (H_2_O) was evaporated at room temperature. TEM images were analyzed with J-Image software (Java, National Institute of Health (NIH), UK) in order to calculate the particle size distribution of the dried NPs.

#### 2.2.4. Electrophoretic Mobility

The electrophoretic mobility of the particles was measured by the PALS (Phase Analysis Light Scattering) technique using Nano-ZS instrument from Malvern Instruments, Malvern WR14 1XZ, UK. The suspensions must be dilute enough for this technique, and 0.1% *w/v* was used in all cases. pH adjustment was carried out by dropwise addition of KOH (10 mM or 100 mM) or HNO_3_ (same concentrations). Repeated measurements (at least 5 runs were performed) were taken, and the average and standard deviation were taken as representatives of the mobility of each sample.

#### 2.2.5. X-Ray Diffraction

A crystallographic study of both kinds of nanorods was performed on a Bruker D8 Discover diffractometer (Madison, WI, USA), using Cu-Kα. Measurements were performed in the 2*θ* range 4°–53° at 0.02° steps. X-ray diffraction (XRD) patterns were compared to the COD standard data [39] in order to confirm the crystal structure of the products. 

#### 2.2.6. Fourier Transform (FTIR) Infrared Characterization

A Jasco 6200 FT IR spectrometer (Jasco, Tokio, Japan) was used in the Attenuated Total Reflection (ATR) mode for obtaining the IR spectra of the particles and their coatings. The wavenumber range was 400–4000 cm^−1^, and the spectra were obtained at room temperature with 4 cm^−1^ resolution.

#### 2.2.7. Thermogravimetric Analysis

A Shimadzu TGA 50 H (Shimadzu Corporation, Tokyo, Japan) thermogravimetric analyzer (temperature range: ambient to 1500 °C; sensitivity of mass loss 1 μg) was used for the evaluation of the organic vs inorganic mass fraction of the particles. The temperature was raised up to 900 °C at a rate of 10 °C/min in a nitrogen atmosphere (50 mL/min N_2_ flow).

#### 2.2.8. Magnetic Hyperthermia (MH) Response 

The magnetic hyperthermia behavior was determined in a homemade alternating current generator previously described by the authors in references [16,40,41,42]. Briefly, the samples were placed in 2-mL screw vials located in the center of the coil (dimensions of the coil: 20 mm diameter and 45 mm length; number of turns: 8) and insulated from it by means of a styrofoam container. The coil was made of 6 mm refrigerated copper tube connected to an oscillator, in parallel with different capacitor combinations so that the current frequencies could be selected out of 185, 206, 236 and 285 kHz, with 7 A maximum current, and 16.2 kA/m maximum field (or magnetic field induction up to 20.3 mT in air). The field was measured in the center of the coil with a NanoScience Laboratories Ltd. (Staffordshire, UK) magnetic field probe.

Upon switching on the field, the samples increased their temperature at a rate d*T*/d*t*, determined by means of an optical-fiber thermometer (Optocon AG, Dresden, Germany). The initial temperature selected for the sample was the physiological one, 37 °C (this was also the temperature of the pumped refrigerating water), and the concentration of particles in the vial was 10 mg/mL in all cases. With this information, it was possible to evaluate the Specific Absorption Rate (*SAR*), which was obtained using typically the initial slope (up to 30 s) of the *T* vs *t* dependence recorded, as follows:

(1) $$SAR=\frac{CV_{s}}{m}\frac{dT}{dt}$$

where *C* is the volume heat capacity of the sample ($C_{H_{2}O}=4185$ J/LK), vs is the sample volume (0.5 mL in the reported experiments), and *m* is the mass of magnetic solids in the sample (5 mg). The origin of the heating is briefly revised in the Supplementary Materials.

#### 2.2.9. Doxorubicin Loading and Release

DOX was incorporated onto both kinds of MNRs by dispersing them in an aqueous solution of the drug. An amount of 1 mL of DOX solution in PBS (200 µM) was mixed with 10 mg of dried functionalized magnetic nanorods and this mixture was stirred for 18 h at 25 °C. DOX-nanorods were then magnetically decanted, washed 3 times with distilled water and freeze-dried. Supernatants were used to determine the non-adsorbed amount of DOX: for that purpose, their optical absorbance at 489 nm was measured in a Jenway 6705 (Cole-Parmer Ltd., Staffordshire, UK) UV–Vis spectrophotometer. Using a calibration line (absorbance vs DOX known concentration), the drug concentration could be obtained by interpolation. The same instrument was used for calculating the amount of DOX desorbed. In this case, 20 mg of the drug-loaded particles was dispersed in a 5 mL volume of the PBS buffer, and kept under agitation. At a given time, the magnetic NPs were decanted by application of a 500 mT permanent magnet, and 0.5 mL of the supernatant was pipetted off, and its absorbance measured after centrifugation at 20,000 rpm for 30 min (in order to get rid of any small amount of MNPs remaining after magnetic decantation): this allowed us to know the amount of DOX released until that time. The volume of the suspension was replenished by adding 0.5 mL PBS and the process continued until the next extraction.

The release study was carried out both in the absence of magnetic field (keeping, in this case, the temperature at 43 °C with a thermostatting bath), and with the AC magnetic field applied. In the latter experiments, two different pHs were tested, and the magnetic field strength (the current through the coil) was controlled in order to ensure a constant temperature of 43.0 ± 0.5 °C.

## 3. Results and Discussion

### 3.1. Morphology and Particle Size Distribution

Well-defined MNRs were obtained with both synthesis methods (hydrothermal –H2M- and co-precipitation –J3M). In the case of hydrothermal synthesis, the analysis reported by Ocaña et al. [43] confirms that phosphate ions are ultimately responsible for the spheroidal shape, as they adsorb on planes parallel to the *c*-axis of growing hematite nanocrystals, facilitating their growth only in the *c*-direction, this providing the anisotropic shape. When the co-precipitation method was used, advantage was taken of the role of PEI as capping agent in the growth of β-FeOOH nuclei. According to Mohapatra et al. [38] and Mozo et al. [44], PEI adsorbs onto (200) planes of the growing nanocrystals, forcing again the anisotropic growth.

Table S1 of the Supporting Information file shows how the synthesis conditions affect the MNRs size (see also Figure S1): briefly, a longer reaction time allowed the obtaining of smaller MNRs. Temperature also affects the morphology and the particle size: for the same reaction time, a higher temperature produced smaller MNRs up to 250 °C. Above this temperature, the rod-like shape was lost. In the co-precipitation approach, a precipitating agent was needed to produce the magnetite or maghemite nanorods. PEI was used for this purpose, and to control the pH of the synthesis medium, a very important issue in achieving a homogeneous morphology and particle size distribution [45]. Depending on the amount of precipitating agent used (Table S2), it is possible to control the particle size distribution of the MNRs (Figure S2): the higher volume of PEI used in the rod preparation produced a shorter length of Fe_2_O_3_ NRs and a more heterogeneous particle size distribution.

**Figure 1:**
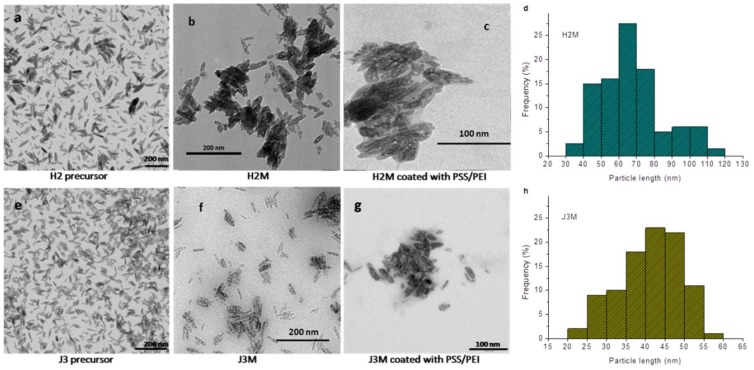
TEM images of the precursor hematite rods (**a**,**e**), the two kinds of magnetic nanoroads (MNRs) (**b**,**f**), and the polymer coated MNRs (**c**,**g**). Length histograms obtained from the TEM images of the MNRs using J-image software are shown on panels (**d**,**h**). H2 obtained by hydrothermal method, and J3 by co-precipitation.

Both synthetic methods started with the preparation of hematite precursors; as illustrated in Figure 1a,e, these are homogeneous, well-dispersed, rod-shaped particles. The shape was maintained after reduction of the precursors in the oven at 300 °C, yielding the desired MNRs (Figure 1b,f). Polymer coating was also clearly observed by TEM in both MNRs (Figure 1c,g). The histograms of the particle length distributions deduced from the TEM images reveal a mean of 64 ± 20 nm particle length for H2M MNRs (Figure 1d), and 45 ± 11 nm for J3M (Figure 1h). In general, the hydrothermal method requires longer reaction times to achieve particle sizes under 100 nm [46]. For our reaction conditions, 10 days were needed to get a mean particle size of 64 nm. Shorter reaction times produced larger particle sizes (Figure S1). The co-precipitation method can be used to produce smaller particle sizes in shorter periods of time, as shown in Figure 1.

### 3.2. Electrical Characterization: Isoelectric Point Determination, Polymer Coating and Its Stability

**Figure 2:**
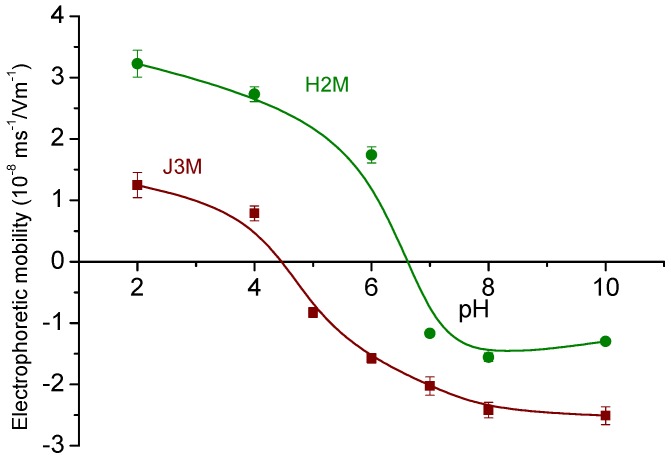
Electrophoretic mobility of the synthesized MNRs in 1 mM NaCl as a function of pH.

The synthesized MNRs were analyzed by electrophoresis in order to study their stability for different pHs of the medium (Figure 2). The isoelectric points (pH_iep_) were obtained for both MNRs in 1 mM NaCl solutions; their values (6.6 for H2M, and 4.5 for J3M) are in a good agreement with those reported in literature for magnetite [47] and maghemite [48], respectively.

These studies can be considered as preliminary for evaluating the proper media conditions to carry out the polymer coating according to the Layer-by-Layer electrostatic self-assembly (LbL-ESA) technique. The adsorption of the first polymer layer (PEI) was carried out starting with an aqueous MNRs suspension at pH 8, in order to ensure that the particles were negatively charged. After PEI adsorption, the MNRs shifted their surface charge to positive, and the second polymer layer (PSS) could be added. Electrophoretic mobility data, as shown in Table 1, provide a follow-up of the successive stages.

In order to test the stability with time of the polymer layers in aqueous media, electrophoretic mobility measurements were repeated for 28 days on the coated samples. Before measuring, the MNRs were magnetically decanted, the supernatant was removed, and fresh water was used each day to re-disperse the MNRs. As observed in Figure S3, the mobility of the two kinds of PEI/PSS-coated particles decreased only slightly during the measurement period, indicating a very limited loss of PSS molecules with time.

### 3.3. XRD Diffraction 

Nine characteristic peaks of hematite (at 2*θ* ≈ 24.2, 33.2, 35.7, 40.9, 49.5, 54.1, 57.7, 62.5 and 64.1 degrees, Cu Kα radiation), marked by their Miller indices ((012), (104), (110), (113), (024), (116), (122/018), (214), (300)), were observed for both precursor samples (Figure S4). After reduction, the precursors transformed into magnetite or maghemite species, as confirmed by the fact that the angles and intensities of the diffraction peaks are consistent with the standard pattern for COD 9009782 (hematite) for both precursors, and COD 9006317 (maghemite) and COD 9010939 (magnetite), for reduced J3M and H2M nanorods [39], respectively. The samples show broad peaks, indicating that the nanorods are polycristalline.

### 3.4. Thermogravimetric Analysis

**Figure 3:**
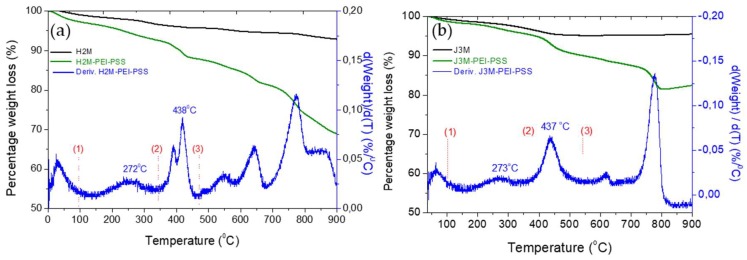
Thermograms of uncoated (**a**) and coated (**b**) magnetic MNRs.

A thermogravimetric analysis was used for the evaluation of the thermal stability of the magnetic particles, as well as to check the polymer/magnetite weight ratio and the polymer components. Figure 3 shows the weight loss of both MNRs before and after polymer coating. Taking into account the inorganic residue of the MNRs before coating (95%), it is possible to calculate the percentage of inorganic component versus the organic ones in the thermogram of the layered MNRs, obtaining a 92% inorganic component (maghemite) for J3M MNRs and 89.6% magnetite for H2M MNRs. 

The first derivative of the weight loss curve of the polymer-coated MNRs shows the degradation temperature of the different components. The first weight loss below 100 °C belongs to water molecules entrapped in the polymer matrix coating the MNRs. The second peak at 273 °C, corresponds to the degradation of the PSS [49], while the third peak at 437 °C can be attributed to the degradation of PEI [50]. Finally, the weight loss between 600 and 800 °C was due to the reduction of magnetite to α-Fe and FeO species (in the case of J3M maghemite degrades first to magnetite [51]). Once the degradation peaks were identified in the thermogram, it was possible to quantify the percentage of the organic components for both MNRs. Details are provided in Table 2. In both MNRs the magnetic component was nearly 90%, which secures the optimal response under a magnetic field and therefore, a proper behaviour for hyperthermia applications.

### 3.5. ATR-FTIR Characterization

The ATR-FTIR spectra are shown in the Figure S5, and they show evidence of the polymer coating on both kinds of particles. The PEI characteristic bands (Figure S5) at 1043–1355 cm^−1^ correspond to stretching vibrational modes of C–N bonds, those at 1720–1780 cm^−1^ belong to the imide group, 5 and at 3100–3300 cm^−1^, we can observe the vibration of the N–H bonds [4]. PSS shows its characteristic peaks at 600–900 cm^−1^ (C–S) and 1050–1200 cm^−1^ (S=O) due to the sulfonate group, and at 1600–1680 cm^−1^ due to the aromatic ring (C=C). Both polymers are in aqueous solution, hence the intense band at 3200–3500 cm^−1^, corresponding to the OH groups of water. Magnetite and maghemite show their Fe–O vibration peaks at 629 cm^−1^.

### 3.6. Magnetic Hyperthermia

**Figure 4:**
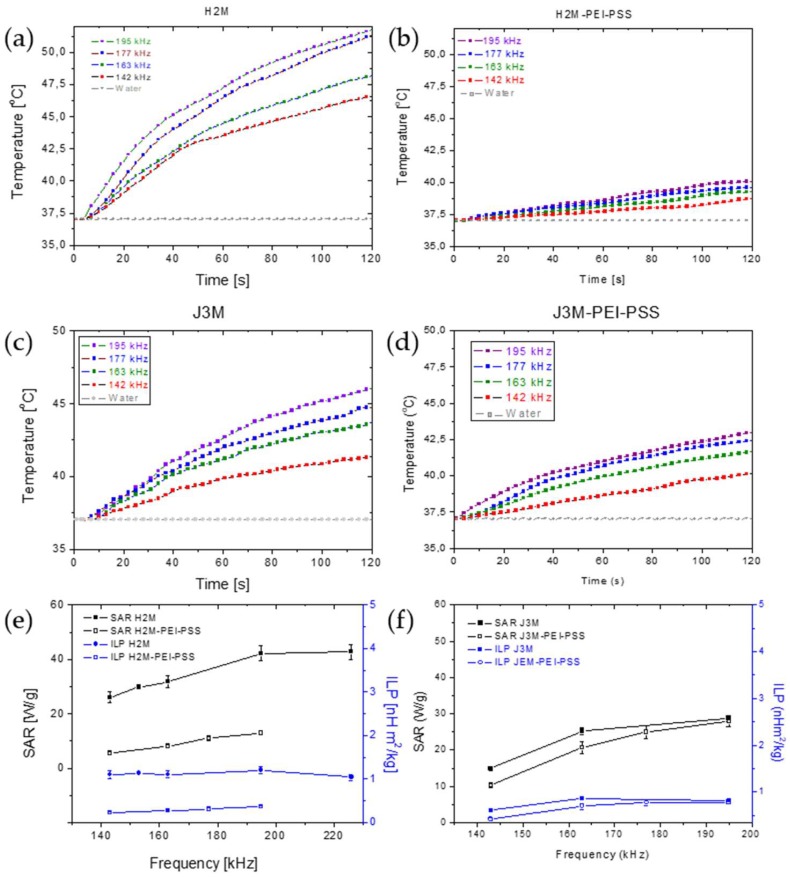
Hyperthermia response tests: temperature-time curves of uncoated and coated magnetic MNRs (**a**–**d**). SAR and ILP values vs. frequency (**e**,**f**).

As mentioned, the hyperthermia response, as measured by the *SAR* value, depends on the morphology and anisotropy of the particles. Other authors have demonstrated, for example, that cubic or octopods iron oxide nanoparticles had superior magnetic heating efficiency as compared to spherical particles of similar sizes [52,53,54], especially in the high field region. It has also been reported that the formation of spherical particle chains increased the *SAR* over that reached by individual separated particles [55]. Figure 4 shows the temperature-time plots of both bare and coated magnetic MNRs at different frequencies of the alternating current (ac) field, as well as the *SAR* and *ILP* reached with each type of particles. As observed, longer MNRs (64 nm, H2M) showed higher *SAR* than MNRs of 40 nm length (J3M) (Table 3). *ILP* values in both magnetic MNRs were larger than in most spherical magnetic nanoparticles, with a similar particle size, described in literature [41,56,57]; this is the advantage of using rod-shaped magnetic nanoparticles in hyperthermia with a higher shape and magnetic anisotropy and therefore, a greater coercivity. In fact, the larger *SAR* values found for non-spherical particles have been found for geometries other than spheroidal. A comparison recently carried out by Nikitin et al. [58] between the hyperthermia performance of spherical, cubic and rod-like MNPs demonstrated that the latter yield the highest value, with *SAR* above 800 W/g, which the authors assigned to the high coercivity of the particles, and Albarqi et al. [59] used hexagonal MnCo iron oxides capable of reaching *SAR* values in excess of 500 W/g for hyperthermia treatment of ovarian cancer cells. All these results point to the extreme interest of non-spherical MNPs in hyperthermia, and the contributions of Noh et al. [60] regarding the basis of nanoscale magnetism indicate that the increased coercivity of the MNPs was on the basis of this extremely good performance.

However, when magnetic MNRs were coated, the shorter MNRs showed a slightly higher hyperthermia response, with a mean SAR, between 10 and 25 W/g and an ILP of 0.29 nHm^2^/kg for J3M-PEI-PSS, as compared to 6–13 W/g and 0.24 nHm^2^/kg for H2M-PEI-PSS (Table 3). As expected, the reduction in the relative mass of magnetic material in the coated rods led to a decrease in heat release per unit mass of particles. Nevertheless, this decrease was smaller in J3M MNRs, probably due to the smaller particle size of these particles and the thinner polymer coating on them, as determined by composition analysis obtained by TGA characterization. In general, SAR and ILP values are independent of the frequency used to apply the ac field, specially at high frequencies (Figure 4e,f).

### 3.7. Doxorubicin Loading and Release

**Figure 5:**
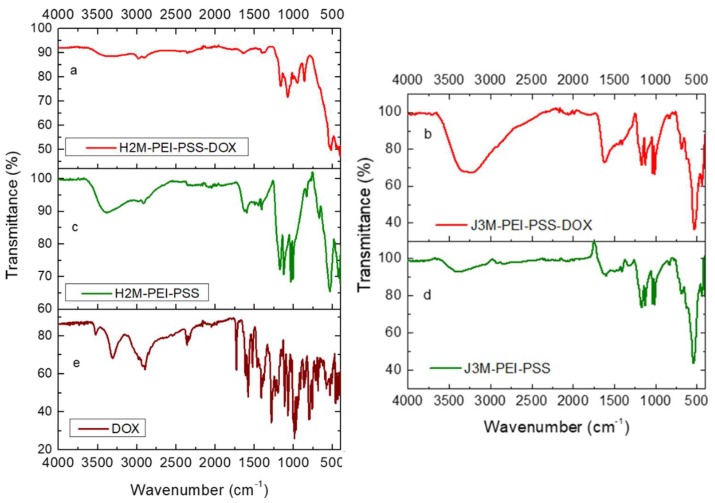
FTIR spectra of the DOX-loaded (**a**,**b**) and polyelectrolyte coated (**c**,**d**) magnetic MNRs and of pure DOX (**e**).

The adsorption of DOX on the MNRs was qualitatively evidenced by electrophoretic mobility data, as depicted in Table 1. A FTIR analysis was also applied to confirm the loading. Figure 5 shows the FTIR spectra of the coated magnetic MNRs modified after the adsorption of doxorrubicin and compared to that of pure DOX and coated particles. The adsorption of DOX onto the coated magnetic MNRs resulted in a change of the FTIR spectrum, as shown in Figure 5a: additional bands that can be ascribed to doxorubicin appeared at 990–1120 cm^−1^ and 1210–1290 cm^−1^. Methylene stretching vibrations at 2850 and 2910 cm^−1^, methyl stretching and aromatic C−H stretching at 2940 and 3030 cm^−1^, as well as amine stretching at 3340 cm^−1^ (DOX) were merged into a very broad band in the 2800−3600 cm^−1^ range, most probably due to the intermolecular hydrogen bonds between the polymers and adsorbed doxorubicin.

However, neither electrophoresis nor FTIR allow the quantification of the percentage of adsorbed drug. For this purpose, UV–visible spectroscopy was used, as it made it possible to quantify the amount of drug adsorbed and released as a function of time. Table 4 shows the results of DOX loading for both magnetic MNRs. It can be observed that the amount of adsorbed drug was higher for the smaller MNRs (J3M-PEI-PSS) as compared to the larger (H2M-PEI-PSS) ones. This may be a result of the larger surface area of the former particles, but additionally, the J3M-PEI-PSS particles have a larger negative surface charge (Table 1), and this favors adsorption of the positive DOX molecules by electrostatic interaction.

Recall that the DOX molecule shows an amino group in its structure, which presents a p*K_a_* of 9.93 [61]. When this drug is in salt form (DOX-HCl) it can be dissolved in water, and it dissociates into doxorubicin, H^+^, and Cl^−^. The primary amine of doxorubicin is then positively charged at acidic pH, which makes the molecule more soluble. However, the carbonyl group of C13 in DOX can be negatively charged at pH > 8, and therefore, the total surface charge of DOX at pH 7.4 is slightly positive or close to neutral. Then, at physiological pH, the release of DOX is slow due to the limited ionization of the drug, which makes it less water soluble. Nevertheless, at acid pH (pH = 5.5), DOX is highly positively charged and therefore, very water soluble. For this reason, the DOX release is expected to be faster at acid than at physiological pH, as was proven in the release test (see below).

The release of the drug was investigated in three experimental conditions: maintaining the solution at 43–44 °C, releasing while applying hyperthermia at pH 5.5, and repeating the latter procedure at pH 7.4. In these cases, the MNRS loaded with DOX were exposed to the magnetic field until they reached the desired temperature of 43–44 °C, typical for hyperthermia-induced apoptosis. After a rapid increase in temperature, the system was stabilized at 43–44 °C by controlling the ac current applied to the coil.

**Figure 6:**
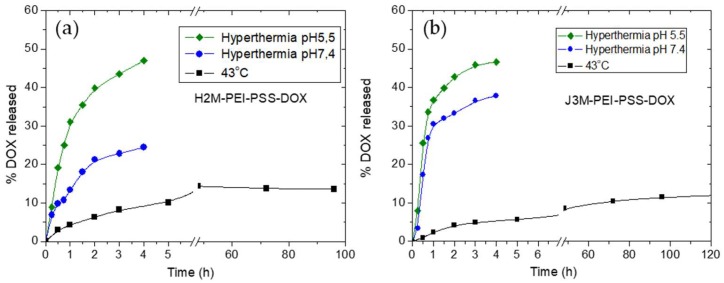
DOX release at 43 °C without the application of AC field (squares) (**a**), under AC field (**b**) at pH 7.4 (circles), and under AC field at pH 5.5 (diamonds), from both coated magnetic nanorods.

As can be observed in Figure 6, the amount of drug released after 4 h at 43°C without the ac field applied was 10% for H2M-PEI-PSS MNRs and 8% for J3M-PEI-PSS MNRs. These percentages of DOX release did not increase markedly after 120 h, probably due to the poor DOX solubility at physiological pH. Conversely, during application of the ac field (hyperthermia-triggered release), the DOX released was increased to a great extent, reaching 25% and 40% of DOX released for H2M-PEI-PSS MNRs and J3M-PEI-PSS MNRs, respectively, at physiological pH after the first 4 h. The response of release to hyperthermia was also analyzed at acid pH (pH 5.5), where the DOX shows better water solubility. Here, the release of the drug achieved 50% after 4 h for both MNR systems. These results prove that the application of an ac field significantly improved the release of DOX, allowing for a significant release of this drug in a local area where the magnetic field was applied.

The faster release with hyperthermia in acid environment is obviously beneficial for cancer treatment. Although the tumor pH may vary according to the tumor area, and the intracellular pH cells within healthy tissues and tumors is similar, tumors exhibit a lower extracellular pH than normal tissues—between 6.0 and 7.0—whereas, in normal tissues and blood, the extracellular pH of is around 7.4 [62]. The low extracellular tumor pH mostly arises from the high glycolysis rate in hypoxic cancer cells. Thus, in the low pH extracellular environment of the cancer cells, DOX can diffuse more easily through the cell membrane and this enhances its role as an anticancer drug.

## 4. Conclusions

Well defined magnetic nanorods (MNRs) were obtained by two synthesis methods, namely, hydrothermal and co-precipitation. The latter allows for the preparation of smaller particle sizes in shorter reaction times. The obtained MNRs (magnetite and maghemite) were successfully coated with ionic polymers and loaded with doxorubicin (DOX) using the well-established LbL technique. High drug loading was achieved for both MNRs, which was thus demonstrated to be excellent drug carrier candidates, with the additional advantage of being suitable for remote manipulation when exposed to external magnetic fields. Furthermore, the two formulations, both before and after coating, showed significant hyperthermia when subjected to alternating magnetic fields with a frequency in the 100–200 kHz range and a field strength of 10–20 kA/m. Not only can this magnetic hyperthermia be applied to tumor treatment by controlling the temperature elevation of the tumoral tissue, but it was found that hyperthermia increased the rate of DOX release from the drug-loaded particles, especially in acidic media, where 50% of the loaded DOX could be released from both types of nano-rods particles in less than 4 h.

## Figures and Tables

**Table 1:** Electrophoretic mobility of bare MNRs, and of the same particles after being coated with polyelectrolytes as indicated. The mobility measured after contact with DOX in solution is also included.

Sample	Electrophoretic Mobility (10^−8^ ms^−1^/Vm^−1^)
H2M	−2.23 ± 0.08
H2M-PEI	2.10 ± 0.04
H2M-PEI-PSS	−3.73 ± 0.13
H2M-PEI-PSS-DOX	0.79 ± 0.05
J3M	−2.48 ± 0.08
J3M-PEI	0.10 ± 0.05
J3M-PEI-PSS	−4.8 ± 0.3
J3M-PEI-PSS-DOX	−3.5 ± 0.4

**Table 2:** Mass percentage of the components (inorganic and polymeric) of bare and coated magnetic NRs calculated from TGA curves.

H2M-PEI-PSS	% Weight	J3M-PEI-PSS	% Weight
Fe_3_O_4_	89.6	γ-Fe_2_O_3_	92.0
PEI	5.1	PEI	3.2
PSS	5.3	PSS	4.8

**Table 3:** SAR and ILP values calculated for un-coated and coated magnetic MNRs.

Sample	SAR (W/g)	ILP (nHm^2^/kg)
H2M	26–43	1.12 ± 0.06
H2M-PEI-PSS	6–13	0.24 ± 0.04
J3M	15–26	0.52 ± 0.08
J3M-PEI-PSS	10–25	0.29 ± 0.05

**Table 4:** Quantification of the doxorubicin adsorbed onto the coated magnetic MNRs (drug loading).

Sample	% Adsorbed DOX	µg DOX/mg MNRs
H2M-PEI-PSS	64.4	7.0
J3M-PEI-PSS	84.1	20.0

## Supplementary Materials

The following are available online at https://www.mdpi.com/1999-4923/11/10/517/s1, Origin of the heating by magnetic hyperthermia; Figure S1: TEM images of different hydrothermal synthesis processes for obtaining MNRs, Figure S2: TEM images of different co-precipitation syntheses producing MNRs, Figure S3: Stability test: time evolution of the electrophoretic mobility measurements of polymer-coated MNRs, Figure S4: X-ray diffraction patterns of precursor hematite nanorods, and their magnetic products (magnetite and maghemite), Figure S5: ATR-FTIR spectra of bare and coated H2M and J3M MNRs, and of the individuall coating polymers, Table S1: Synthesis conditions of the hydrothermal process and mean particle length, Table S2: Synthesis conditions of the co-precipitation method and mean particle length of precursor nanorods.
