# Supplementary material for: Hyperthermia-Triggered Doxorubicin Release from Polymer-Coated Magnetic Nanorods
Source: Pharmaceutics. 2019 Oct 8;11(10):517. doi: 10.3390/pharmaceutics11100517 (PMC6835812; doi:10.3390/pharmaceutics11100517)
Supplement: Supplementary file 1 [file pharmaceutics-11-00517-s001.pdf]

pharmaceutics

Supplementary Materials: Hyperthermia-Triggered Doxorubicin Release from Polymer-Coated Magnetic Nanorods

Felisa Reyes-Ortega, Blanca Luna Checa Fernández, Angel V. Delgado and Guillermo R. Iglesias *

1. Origin of the Heating by Magnetic Hyperthermia

The heating induced by application of an alternating magnetic field can be understood using the simple approach known as linear-response theory, according to which, even if the particles are assumed to be immobile in a non-magnetic matrix, hysteresis in the magnetization M vs magnetic field H cycle will be present, related to the process known as Néel-Brown relaxation, due to the finite time required for inversion of the particle's magnetic moment orientation when the field changes direction (1–3). In actual hyperthermia applications, the particles are not immobile in rigid matrix, but they can rather oscillate back and forth, following the field harmonic oscillations. Viscous friction with the dispersion medium produces again a delay between magnetization and field, and a subsequent source of hysteresis, and hence heating of the system. This is the so-called Brownian relaxation. Applying a field with a frequency close to the reciprocal of the characteristic time of the process involved (mainly Brownian in many practical cases) maximizes the imaginary component of the magnetic susceptibility, χ'', and, as a consequence, the area (energy released per unit volume and per cycle) of the hysteresis cycle. In fact, in the context of the simple linear model, it can be demonstrated that the rate of heat release is proportional to χ'', as well as to the frequency and squared amplitude of the field, H_0. *SAR* values are usually in the order of tens to hundreds of W/g. If one wishes to compare the hyperthermia efficiency of different materials, no matter the device used, it is interesting to define a new quantity known as *Intrinsic Loss Power* (*ILP*), approximately free of the experimental details. This is given as:

$$ILP = \frac{SAR}{fH_0^2} \tag{1}$$

with typical values in the order of 10^{-9} Hm^2 kg^{-1}.

2. Synthesis Conditions

Table S1. Synthesis conditions of the hydrothermal process and mean particle length.

Sample	Synthesis Temperature (°C)	Synthesis Reaction Time (h)	NRs Mean Length (nm)
**H1**	160	12	430 ± 80
**H2**	100	240	64 ± 20
**H3**	200	12	390 ± 60
**H4**	250	12	330 ± 40
**H5**	300	12	No nanorod shape
**H6**	100	12	530 ± 60

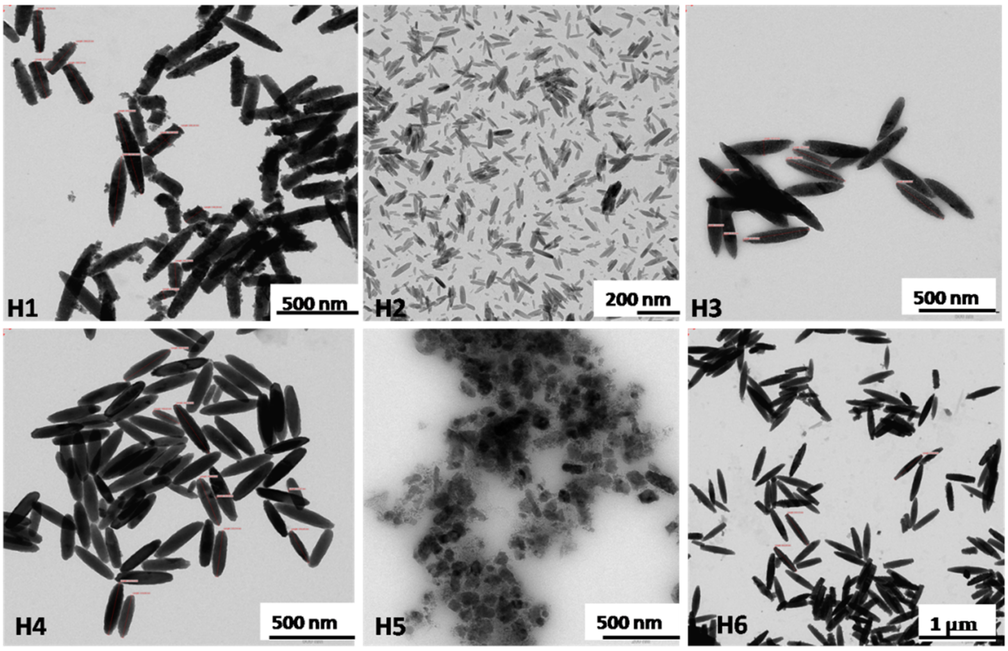

Figure S1. TEM images of different hydrothermal synthesis processes for obtaining MNRs.

Table S2. Synthesis conditions of the co-precipitation method and mean particle length of precursor nanorods.

	Precipitating Agent (PEI) Added (mL)	Fe_2O_3 NR Length (nm)
**J1**	2	16 ± 3
**J2**	1	39 ± 14
**J3**	0.5	45 ± 11

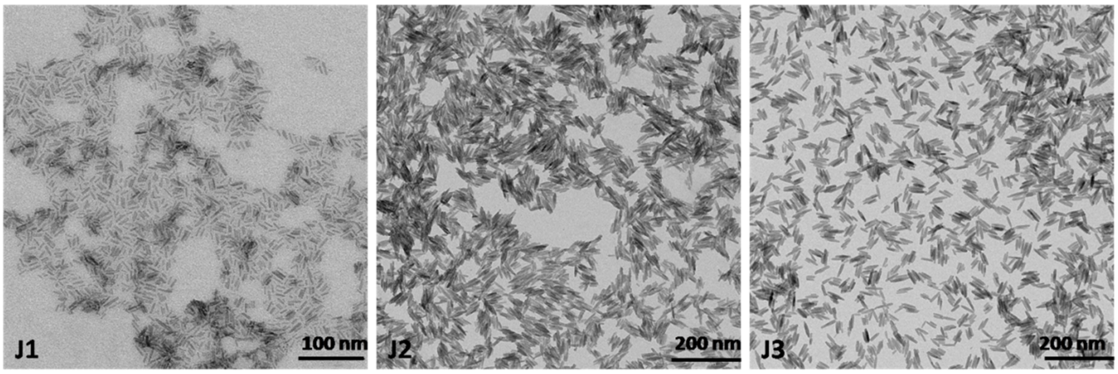

Figure S2. TEM images of different co-precipitation syntheses producing MNRs.

3. Stability as Evaluated by Electrophoresis

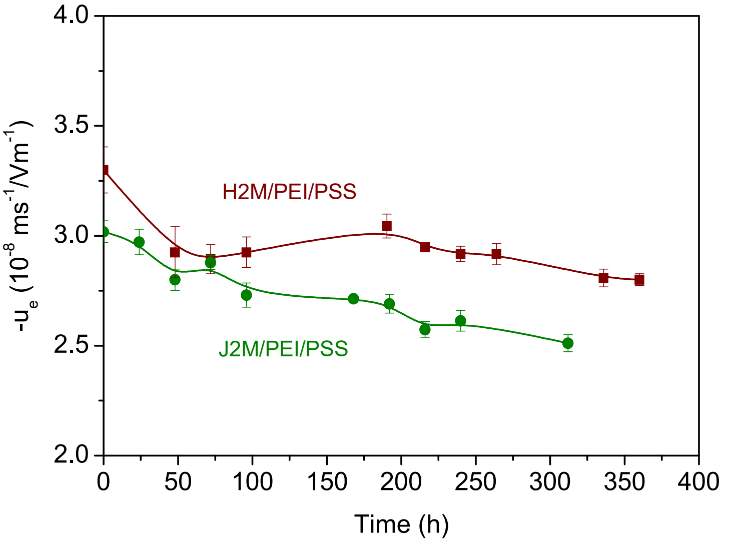

Figure S3. Stability test: time evolution of the electrophoretic mobility measurements of polymer-coated MNRs.

4. X-Ray Diffraction Patterns

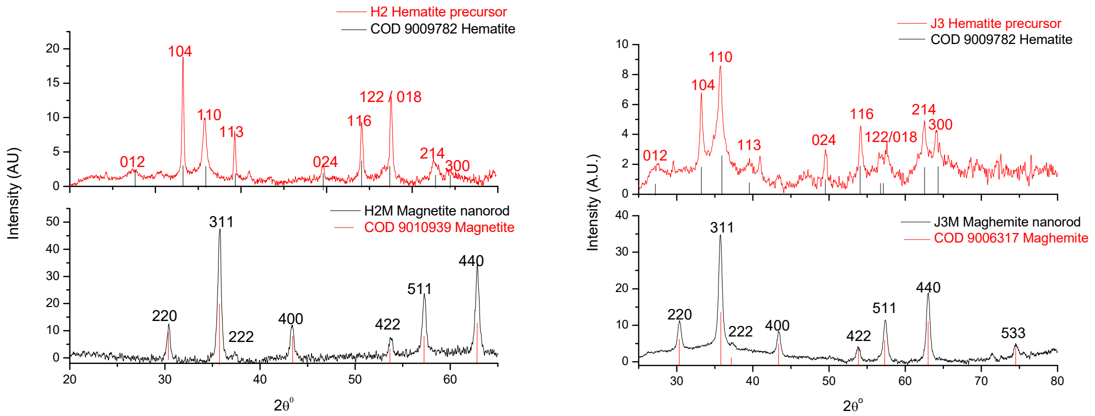

Figure S4. X-ray diffraction patterns of precursor hematite nanorods, and their magnetic products (magnetite and maghemite).

5. ATR-FTIR Characterization

The PEI characteristic bands (Figure S5) at 1043–1355 cm^{-1} correspond to streching vibrational modes of C–N bonds, those at 1720–1780 cm^{-1} belong to the imide group, and at 3100–3300 cm^{-1} we can observe the vibration of the N–H bonds (4). PSS shows its characteristic peaks at 600–900 cm^{-1} (C–S) and 1050–1200 cm^{-1} (S=O) due to the sulfonate group, and at 1600–1680 cm^{-1} due to the aromatic ring (C=C). Both polymers are in aqueous solution, hence the intense band at 3200–3500 cm^{-1}, corresponding to the OH groups of water. Magnetite and maghemite show their Fe–O vibration peaks at 629 cm^{-1}.

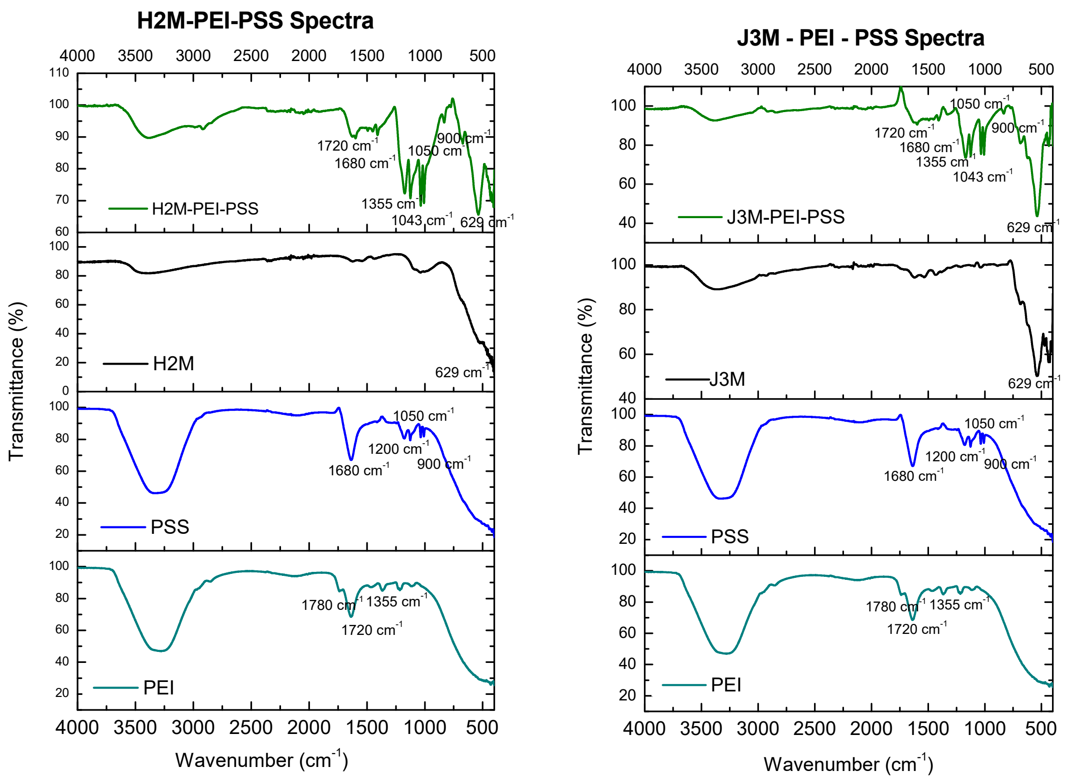

Figure S5. ATR-FTIR spectra of bare and coated H2M (left) and J3M (right) MNRs, and of the individaul coating polymers (bottom).

References

1. Pankhurst QA, Thanh NTK, Jones SK, Dobson J. Progress in applications of magnetic nanoparticles in biomedicine. *J Phys D-Appl Phys* 2009;**42**:224001.
2. Laurent S, Dutz S, Hafeli UO, Mahmoudi M. Magnetic fluid hyperthermia: Focus on superparamagnetic iron oxide nanoparticles. *Adv Colloid Interf Sci.* 2011;**166**:8–23.
3. Ortega D, Pankhurst QA. Magnetic hyperthermia. In: O'Brien P, editor. *Nanoscience Vol. 1: Nanostructures through Chemistry*. Cambridge: Royal Society of Chemistry; 2013, p. 60–88.
4. Djoumessi Lekeufack D, Brioude A, Lalatonne Y, Motte L, Coleman AW, Miele, P. Reversible multi polyelectrolyte layers on gold nanoparticles. *J Nanopart Res* 2012;**14**:1–7.
